# Dual diagnosis at the neuro-immune interface: a case report of neuronal intranuclear inclusion disease with acute anti-CASPR2 encephalitis

**DOI:** 10.3389/fimmu.2025.1650420

**Published:** 2025-08-26

**Authors:** Wan Zhu, Tiansi Liu, Xinran Yu, Min Peng, Jinghan Hu

**Affiliations:** ^1^ Kunming University of Science and Technology, Affiliated Hospital, People’s Hospital of Wenshan Prefecture, Department of Radiology, Wenshan, China; ^2^ Kunming University of Science and Technology, Affiliated Hospital, People’s Hospital of Wenshan Prefecture, Department of Neurology, Wenshan, China; ^3^ The Affiliated Hospital of Kunming University of Science and Technology, Kunming, China

**Keywords:** anti-CASPR2 antibody encephalitis, autoimmune encephalitis, Morvan syndrome, neuronal intranuclear inclusion disease, *NOTCH2NLC*

## Abstract

Neuronal intranuclear inclusion disease (NIID) is a rare autosomal-dominant, progressive neurodegenerative condition characterized by complex and variable clinical manifestations that can affect multiple neurological domains. This report describes the case of a 49-year-old female patient with a 10-year history of headaches, whose older sister had been diagnosed with NIID 1 year earlier through genetic testing and a skin biopsy. Recently, the patient developed dizziness and vomiting. Although symptomatic treatment reduced incidents of vomiting, her dizziness progressively worsened. It was accompanied by lower limb weakness, gait instability, hallucinations, and abnormal sleep behaviors. Routine imaging and cerebrospinal fluid immunological and microbiological tests revealed no abnormalities. Genetic analysis revealed a 130-repeat expansion in the *NOTCH2NLC* gene, and skin biopsy confirmed the presence of intranuclear inclusions, establishing the NIID diagnosis. However, during hospitalization, the patient’s bilateral lower limb tremors, hallucinations, and abnormal sleep behaviors suggested a possible acute encephalitic process. Subsequent serum testing detected positive anti-contactin-associated protein-like 2 (CASPR2) antibodies. Significant symptom improvement following treatment with immunoglobulins and steroids supported the diagnosis of coexisting acute anti-CASPR2 antibody encephalitis and NIID. This is the first reported case of dual disease coexistence.

## Introduction

When neurodegenerative diseases coexist with autoimmune diseases exhibiting similar clinical symptoms, diagnosis and treatment present significant challenges. The boundaries between neurodegeneration and autoimmunity are increasingly blurred, with growing evidence suggesting that immune mechanisms play a significant role in traditionally defined neurodegenerative conditions (1). Neuronal intranuclear inclusion disease (NIID) is a progressive autosomal dominant neurodegenerative disorder previously thought to be almost exclusive to Asian populations, but now identified in other ethnic groups (2). The discovery of unstable cytosine-guanine-guanine (GGC) repeat expansions in the 5’ untranslated region (5’-UTR) of the *NOTCH2NLC* gene has established NIID as a novel polyglycine (polyG) disease. Its pathogenesis involves both RNA and protein toxicity (3). Clinically, NIID exhibits significant heterogeneity, manifesting as cognitive impairment, autonomic dysfunction, movement disorders, episodic neurogenic events, and neuromuscular syndromes (4). This diversity has led researchers to propose a classification system of five clinical subtypes based on the most prominent features (4). Characteristic magnetic resonance imaging (MRI) findings aid diagnosis, such as high signal intensity at the corticomedullary junction on diffusion-weighted imaging (DWI), whereas detection of eosinophilic p62-positive intranuclear inclusions in skin biopsy specimens provides definitive confirmation (4). In contrast, anti-contactin-associated protein-like 2 (CASPR2) antibody-mediated encephalitis is a well-defined type of autoimmune encephalitis (AE) that predominantly affects elderly men and presents with subacute-onset neurological symptoms (5, 6). Its core mechanism involves IgG4 subclass-dominant autoantibodies that do not cause target antigen internalization but functionally disrupt the critical interaction between CASPR2 and its binding partner contactin-2 (5). This disruption is believed to impair voltage-gated potassium channel clustering, leading to neuronal hyperexcitability (5, 6). The clinical spectrum of anti-CASPR2 autoimmunity is broad, including limbic encephalitis, peripheral nerve hyperexcitability (neuromyotonia), cerebellar ataxia, and prominent autonomic dysfunction and insomnia (which may manifest as Morvan syndrome) (6, 7). Paroxysmal episodic ataxia is also a recognized but less common feature (7). Unlike NIID, anti-CASPR2 encephalitis typically responds to immunotherapy, making accurate and timely diagnosis crucial for improving patient outcomes (8, 9).

Advances in genetic and immunological testing have revealed that these once-distinct disease categories may coexist, creating complex clinical presentations that require refined diagnostic and management strategies. Clinically, evidence demonstrates that these two pathological processes may co-occur. For instance, patients with autoimmune encephalitis have been documented to concurrently present with cerebrospinal fluid biomarkers, meeting the criteria for Alzheimer’s disease, specifically low Aβ42 and elevated p-tau (10). While the simultaneous occurrence of a hereditary neurodegenerative disease and an antibody-mediated autoimmune encephalitis is rare, such coexistence underscores the importance of a comprehensive evaluation when symptoms cannot be fully explained by a single pathological mechanism.

Although both NIID and anti-CASPR2 encephalitis can present with overlapping features, such as cognitive decline, seizures, and ataxia, their concurrent diagnosis is extremely rare. This “dual diagnosis” poses significant challenges, requiring clinicians to distinguish symptoms of acute, treatable autoimmune attacks from manifestations of chronic, progressive neurodegenerative processes (4, 5). This study reports a case of genetically and pathologically confirmed NIID in a patient who developed acute encephalitic symptoms, including the characteristic severe sleep disturbances of Morvan syndrome (7), which led to the discovery of coexisting anti-CASPR2 antibody-mediated encephalitis. This case illustrates the intricate intersection of neurodegeneration and autoimmunity and emphasizes the necessity of a comprehensive diagnostic evaluation when clinical symptoms exceed the expected progression of a known underlying disease.

## Case description

The patient, a 49-year-old woman, was initially admitted to the gastroenterology department for persistent nausea and vomiting lasting 5 days. Her medical history included chronic headaches for over 10 years and a previous cholecystectomy. During hospitalization, her gastrointestinal symptoms improved; however, she subsequently developed new neurological symptoms, including dizziness and bilateral lower extremity weakness. Due to these emerging concerns, she was transferred to the neurology department for further evaluation.

A neurological examination revealed the following: a drowsy state with abnormal groping movements; fluent speech upon arousal; preserved cognitive function; spontaneous horizontal nystagmus in both eyes; normal proximal muscle strength in the lower limbs (5/5); and mildly reduced distal muscle strength (4/5), accompanied by decreased muscle tone and tremors in both lower limbs. Coordination tests showed symmetrical and accurate finger-to-nose movements bilaterally, but instability in standing and gait. Lower limb tendon reflexes were brisk (++); bilateral pathological signs were negative; and the autonomic nervous function was intact, with normal bladder and bowel control and no orthostatic hypotension (**Supplementary Video 1**). Her mini-mental state examination (MMSE) score was 27/30, and her Modified Rankin Scale (mRS) score was 3.

**Figure 1:**
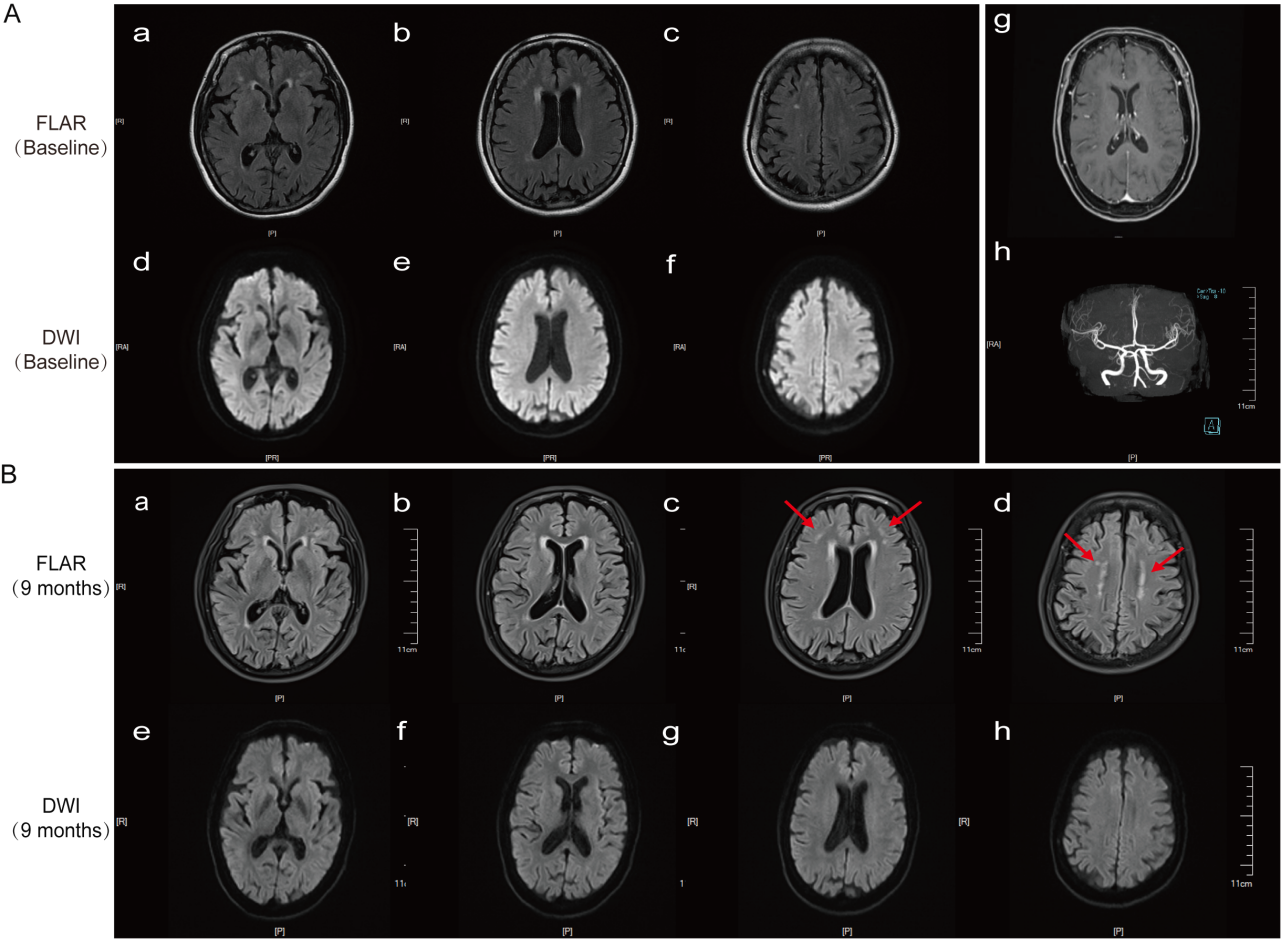
Results of cranial MRI and cerebral artery MRA on 12 June 2024, and follow-up cranial MRI on 20 March 2025. **(A[a–f])** Cranial MRI (DWI) shows bilateral periventricular white matter hyperintensities (WMH) in the frontal and parietal lobes (Fazekas score = 1). **(A[g])** Contrast-enhanced cranial MRI. **(A[h])** Cerebral artery MRA. **(B[a–h])** A follow-up cranial MRI (FLAIR) 9 months later revealed new high signal intensity at the subcortical medullary junction of the bilateral frontal lobes, along with bilateral periventricular WMH (Fazekas score = 2).

The initial head CT scan showed no abnormalities. A 3T MRI scan (including T1, T2, FLAIR, DWI/ADC, contrast-enhanced, and susceptibility-weighted sequences) revealed only mild white matter changes (Fazekas score = 1). No abnormalities were found in whole-brain DWI, ADC, MRA, or contrast-enhanced sequences (**Figure 1A[a–h]**). Further examinations, including EEG, ECG, and echocardiography, all yielded normal results. Laboratory tests, including a complete blood count, inflammatory markers, metabolic parameters, autoimmune screening, and infectious disease testing, showed no significant abnormalities. To rule out encephalitis, additional cerebrospinal fluid tests were performed, including microbiological metagenomic next-generation sequencing (mNGS), autoimmune encephalitis antibodies (including anti-LGI1 and anti-NMDAR), and oligoclonal band testing; all results were negative.

**Figure 2:**
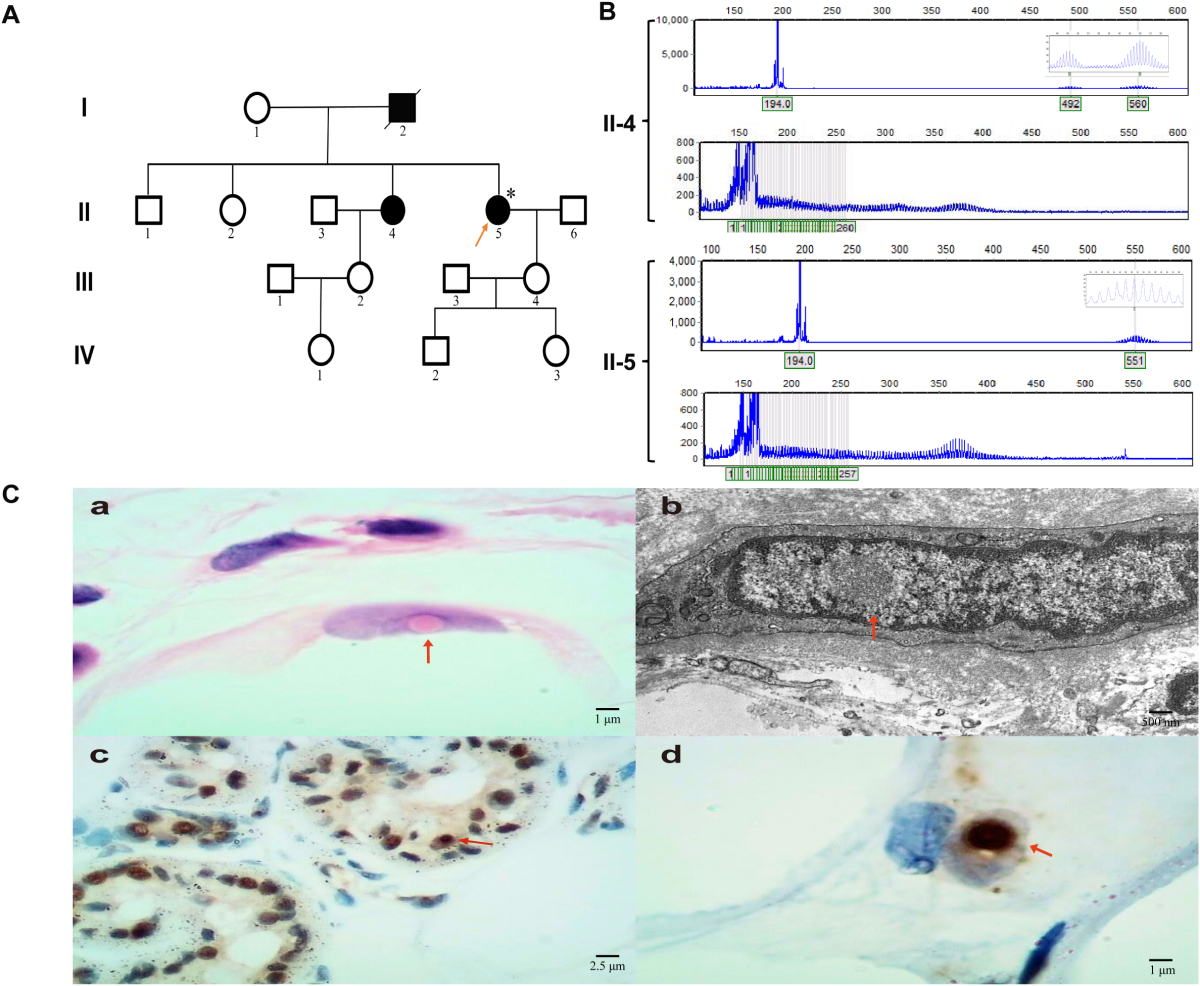
NIID family tree, genetic characteristics of *NOTCH2NLC* repeat expansion, and pathological findings of subcutaneous tissue in the lower leg. **(A)** Pedigree chart. Squares represent male subjects, circles represent female subjects. Filled symbols indicate pathogenic gene carriers, and the arrow points to the proband. **(B)** *NOTCH2NLC* repeat expansion detection chart, with II-4 as the patient’s sister and II-5 as the proband. **(C[a–d])** HE staining, electron microscopy, ubiquitin, and p62 immunohistochemistry results of subcutaneous soft tissue in the lower leg, with arrows indicating intranuclear inclusions.

According to the patient’s family history, her sister had been diagnosed with NIID a year earlier. Her sister primarily exhibits progressive cognitive dysfunction and is currently receiving symptomatic supportive treatment, with slow disease progression (**Figures 2A, B[II-4]**). Dynamic mutation testing of the NOTCH2 gene revealed a GGC repeat expansion in the *NOTCH2NLC* gene, with a repeat count of 130 (**Figures 2A, B[II-5]**). A subsequent skin biopsy revealed typical intranuclear inclusions that were positive for p62 and ubiquitin immunostaining (**Figure 2C[a–d]**), confirming the diagnosis of NIID. To make a differential diagnosis, other genetic disorders that can cause similar tremor and ataxia symptoms were also ruled out, including testing for the *FMR1* gene for Fragile X-associated tremor/ataxia syndrome (FXTAS), which was negative. However, the patient’s progressive cerebellar symptoms, bilateral calf muscle tremors, nystagmus, and psychiatric symptoms could not be explained and were inconsistent with the typical clinical progression of NIID. Her daytime sleepiness, hallucinations, and complex nocturnal behaviors were highly suggestive of encephalitis, with features reminiscent of Morvan syndrome.

To further clarify the diagnosis, we repeated cerebrospinal fluid autoimmune antibody testing and added blood antibody testing. One week later, the report showed positive anti-CASPR2 antibodies in the blood (CBA method, titer 1:10; titers≥1:10 are considered weakly positive), confirming a diagnosis of acute-phase anti-CASPR2 antibody-associated encephalitis. Based on the comprehensive test results, the patient was ultimately diagnosed with anti-CASPR2 antibody encephalitis combined with NIID. Since anti-CASPR2 antibody encephalitis is an IgG4-mediated disease, rituximab (RTX) is considered the treatment of choice for encephalitis symptoms. However, due to a current drug shortage at the hospital, we opted for intravenous immunoglobulin (IVIg) therapy (400 mg/kg/day for 5 days) (11) after excluding an underlying thymoma via a chest CT. Two days after completing the IVIg course, we initiated methylprednisolone sodium succinate pulse therapy (500 mg/day), halving the dose every 3 days until it was reduced to 60 mg, followed by sequential oral steroid therapy. Two weeks later, the patient’s symptoms significantly improved: dizziness lessened; mood and energy improved; diplopia resolved; gait stabilized; muscle strength recovered; and sleep quality improved, with only occasional sleep-talking episodes.

At discharge, the patient was prescribed 60 mg of oral prednisone acetate daily, with a planned weekly reduction of 5 mg. At the 9-month follow-up, serum CASPR2 antibodies were negative; however, a cranial MRI revealed new FLAIR hyperintensities at the corticomedullary junction, predominantly involving the frontal and parietal lobes, suggesting that the patient’s imaging findings were progressively evolving toward a typical NIID presentation (4) (**Figure 1B[a–h]**). A follow-up neurological examination showed clear consciousness, fluent speech, and normal muscle strength and tone in all four limbs, but a bilateral upper limb tremor was noted. The MMSE score was 28/30, the Montreal Cognitive Assessment (MoCA) score was 26/30, and the mRS score was 1. Thereafter, the patient’s clinical symptoms stabilized, and no further signs of encephalitis were observed.

## Discussion

This case exemplifies the profound difficulties in diagnosis and treatment when a hereditary neurodegenerative disorder coexists with autoimmune encephalitis. The patient’s presentation was a clinical puzzle: a background of chronic, subtle neurological symptoms consistent with her genetic predisposition to NIID was acutely overshadowed by a subacute encephalitic syndrome. Considering her decade-long history of headaches and bilateral limb tremors, these symptoms did not fully align with the typical chronic and progressive characteristics of NIID. Although she lacked the typical manifestations described in large cohort studies—such as cognitive decline, movement disorders, or autonomic dysfunction (12)—and her cranial MRI showed no significant abnormalities, including no typical subcortical–medullary junction white matter hyperintensity changes on T2-FLAIR or DWI (4, 13, 14), further genetic testing confirmed a *NOTCH2NLC* GGC repeat expansion (130 repeats) (13, 14), and a skin biopsy revealed intranuclear inclusions with positive p62 and ubiquitin immunohistochemical staining, thus confirming the diagnosis of NIID (3, 15). In cases of atypical clinical presentations, a comprehensive and systematic examination and evaluation can help reduce misdiagnosis.

The diagnostic journey was complex. Initially, the patient’s chronic headaches and tremors were considered atypical manifestations of NIID, especially given her confirmed *NOTCH2NLC* gene expansion and positive skin biopsy results. However, the acute onset of severe encephalopathy, cerebellar ataxia, and particularly, the severe sleep disturbances and hallucinations—suspected to be manifestations of Morvan syndrome—presented a clinical picture that could not be explained by the typical, slowly progressive course of NIID (13). This clinical incongruity prompted a search for an alternative diagnosis, leading to the detection of anti-CASPR2 antibodies in the serum. Although the serum titer was low (1:10), a diagnosis of anti-CASPR2 encephalitis was established based on three key pillars of evidence. First, the patient’s clinical syndrome—with its prominent features of encephalopathy, ataxia, and severe sleep disturbance—was highly characteristic of the disease spectrum (6, 7). Second, as demonstrated by Joubert et al. (7), a significant proportion of patients with anti-CASPR2 encephalitis may present with low titers or even exclusively serum-positive antibodies, making the clinical phenotype a crucial diagnostic determinant. Finally—and most decisively—the patient’s dramatic clinical improvement following immunotherapy served as both a therapeutic success and a powerful confirmation of the underlying autoimmune pathology (**Supplementary Video 1**).

The patient’s therapeutic journey and response provide critical insights into managing complex dual-diagnosis cases. The choice of initial immunotherapy, however, involved navigating a significant clinical and immunological challenge. Recent research highlights that anti-CASPR2 antibodies are predominantly of the IgG4 subtype. A pivotal study showed that 100% of diagnosed patients possess IgG4 antibodies, with 63% also co-expressing the IgG1 subtype (6). This finding has profound therapeutic implications, as IgG4-mediated diseases are known to respond poorly to IVIg but respond well to B-cell depleting therapies such as RTX, which is often recommended as a first-line treatment. In contrast, the IgG1 subtype is typically responsive to IVIg.

Why was our patient started on IVIg rather than RTX? The decision was dictated by both a lack of facilities for anti-CASPR2 antibody subtype analysis and the unavailability of RTX at our hospital. In the absence of this specific information, and considering that a majority of patients also have the potentially IVIg-responsive IgG1 subtype, initiating treatment with the widely available and rapid-acting IVIg (400 mg/kg/day for 5 days) was a pragmatic and justifiable first step (9, 11, 16). As might be predicted for an IgG4-dominant process, the patient’s clinical symptoms showed no significant improvement with IVIg. This lack of response served as indirect evidence of a predominantly IVIg-resistant pathology. This prompted an immediate escalation of therapy. Based on evidence from studies such as that of Abbatemarco et al. (8), we administered high-dose intravenous methylprednisolone (500 mg/day). The effect was dramatic and diagnostically confirmatory. After 2 weeks, the patient’s acute symptoms—including encephalopathy, dizziness, diplopia, and severe sleep disturbances—improved significantly, and her gait became more stable. Long-term follow-up powerfully reinforces this dual-diagnosis framework. Nine months after discharge, the symptoms of anti-CASPR2 encephalitis had not recurred, demonstrating a durable response to the steroid treatment. Conversely, the tremors associated with NIID persisted, and a follow-up brain MRI revealed the onset of new white matter lesions (**Figure 1B[a–h]**) characteristic of NIID’s inexorable progression. This case powerfully illustrates that, in patients with a dual diagnosis, treatment targeting the autoimmune inflammation can be highly effective—even when the underlying neurodegenerative disease continues its course. It underscores that even without access to advanced subtype analysis, a logical, sequential immunotherapeutic approach can successfully manage the treatable component of these complex neurological presentations.

The co-occurrence of NIID and anti-CASPR2 encephalitis in our patient is likely a coincidence, but it could also represent a causal pathophysiological cascade in which primary neurodegeneration triggers a secondary autoimmune response. This concept is central to the evolving understanding of neuroimmunology, which recognizes the intricate link between neurodegenerative processes and subsequent neuroinflammation (1). The underlying pathology of NIID, characterized by widespread neuronal stress, apoptosis, and the formation of intranuclear inclusions, leads to significant cellular and tissue damage (13, 14). We hypothesize that this chronic neuronal injury could disrupt the structural integrity of neurons and the blood-brain barrier, leading to the abnormal exposure or release of neuronal antigens that are typically shielded from the immune system. Furthermore, an alternative or complementary hypothesis could involve the primary pathogenic mechanism of NIID itself. The GGC repeat expansion in the *NOTCH2NLC* gene is known to cause RNA toxicity (3). It is plausible that these abnormal RNA transcripts could be recognized by innate immune sensors as damage-associated molecular patterns (DAMPs), directly activating intracellular immune pathways. This could contribute to the chronic pro-inflammatory state that lowers the threshold for a secondary, antigen-specific autoimmune response against proteins such as CASPR2.

In this specific case, the exposed antigen was likely CASPR2—a crucial protein located in the juxtaparanodal region of myelinated axons (5, 16). The persistent damage caused by NIID may have rendered CASPR2 accessible to the immune system, initiating an autoimmune reaction and the production of pathogenic anti-CASPR2 antibodies. This “neurodegeneration-induced autoimmunity” paradigm offers a plausible explanation for the dual diagnosis. The clinical findings strongly support this hypothesis. The successful immunotherapy acted as both a diagnostic and therapeutic trial. By silencing the secondary autoimmune inflammation, it effectively “unmasked” the primary, inexorable progression of NIID. This was unequivocally demonstrated by the follow-up MRI, which, despite the resolution of encephalitic features, revealed the emergence of new, characteristic high-signal lesions on FLAIR at the corticomedullary junction—a hallmark of advancing NIID (13, 14). This case, therefore, serves as a critical reminder for clinicians: when a patient with a presumed neurodegenerative disorder experiences acute or subacute deterioration, a superimposed, treatable autoimmune process must be considered.

Of course, our study has limitations. Due to hospital conditions, patients with sleep disorders resembling Morvan syndrome were unable to undergo polysomnography. Furthermore, at the 3-month post-discharge mark, we contacted the patient by phone to recommend a follow-up visit; however, due to the patient’s remote rural location, with poor transportation, and the absence of family caregivers (who were working away from home), the visit could not be arranged. The follow-up was eventually conducted at the 9-month post-discharge mark, but the lack of continuous longitudinal assessments using standardized functional scales (such as MMSE, mRS, and ADL) limited our ability to comprehensively describe the long-term interactions between the two diseases.

## Conclusion

This case demonstrates that NIID and anti-CASPR2 antibody encephalitis can coexist as distinct conditions, each requiring specific therapeutic approaches. Successful identification and treatment of the autoimmune component significantly improved the patient’s quality of life, despite the persistent NIID symptoms. These findings emphasize the importance of maintaining a broad differential diagnosis when evaluating complex neurological presentations—especially when the clinical course deviates from the expected disease progression.

## Patient perspective

The patient and her family are satisfied with the diagnosis and treatment outcomes.

## Data Availability

The original contributions presented in the study are included in the article/**Supplementary Material**. Further inquiries can be directed to the corresponding author.

## Supplementary material

Supplementary Video 1 The patient’s clinical manifestations: nystagmus, cerebellar ataxia, and complex sleep behaviors, along with post-treatment changes.

## References

[1] Altmann DM. Neuroimmunology and neuroinflammation in autoimmune, neurodegenerative and psychiatric disease. Immunology. (2018) 154:167–8. doi: 10.1111/imm.12943, PMID: 29878338 PMC5980115

[2] Podar IV Gutmann DAP Harmuth F Haack TB Ossowski S Hengel H. First case of adult onset neuronal intranuclear inclusion disease with both typical radiological signs and NOTCH2NLC repeat expansions in a Caucasian individual. Eur J Neurol. (2023) 30:2854–8. doi: 10.1111/ene.15905, PMID: 37271829

[3] Ishiura H Shibata S Yoshimura J Suzuki Y Qu W Doi K. Noncoding CGG repeat expansions in neuronal intranuclear inclusion disease, oculopharyngodistal myopathy and an overlapping disease. Nat Genet. (2019) 51:1222–32. doi: 10.1038/s41588-019-0458-z, PMID: 31332380

[4] Tai H Wang A Zhang Y Liu S Pan Y Li K. Clinical features and classification of neuronal intranuclear inclusion disease. Neurol Genet. (2023) 9. doi: 10.1212/NXG.0000000000200057, PMID: 37090934 PMC10117695

[5] Patterson KR Dalmau J Lancaster E. Mechanisms of Caspr2 antibodies in autoimmune encephalitis and neuromyotonia. Ann Neurol. (2018) 83:40–51. doi: 10.1002/ana.25120, PMID: 29244234 PMC5876120

[6] van Sonderen A Ariño H Petit-Pedrol M Leypoldt F Körtvélyessy P. The clinical spectrum of Caspr2 antibody-associated disease. Neurology. (2016) 87:521–8. doi: 10.1212/WNL.0000000000002917, PMID: 27371488 PMC4970662

[7] Joubert B Gobert F Thomas L Saint-Martin M Desestret V Convers P. Autoimmune episodic ataxia in patients with anti-CASPR2 antibody-associated encephalitis. Neurol Neuroimmunol Neuroinflamm. (2017) 4:e371. doi: 10.1212/NXI.0000000000000371, PMID: 28638854 PMC5471489

[8] Abbatemarco JR Yan C Kunchok A Rae-Grant A. Antibody-mediated autoimmune encephalitis: A practical approach. Cleve Clin J Med. (2021) 88:459–71. doi: 10.3949/ccjm.88a.20122, PMID: 34341030

[9] Nosadini M Mohammad SS Ramanathan S Brilot F Dale RC. Immune therapy in autoimmune encephalitis: a systematic review. Expert Rev Neurother. (2015) 15:1391–419. doi: 10.1586/14737175.2015.1115720, PMID: 26559389

[10] Chang BK Day GS Graff-Radford J McKeon A Flanagan EP Algeciras-Schimnich A. Alzheimer’s disease cerebrospinal fluid biomarkers differentiate patients with Creutzfeldt-Jakob disease and autoimmune encephalitis. Eur J Neurol. (2022) 29:2905–12. doi: 10.1111/ene.15469, PMID: 35735602 PMC9463096

[11] Seery N Butzkueven H O’Brien TJ Monif M. Contemporary advances in antibody-mediated encephalitis: anti-LGI1 and anti-Caspr2 antibody (Ab)-mediated encephalitides. Autoimmun Rev. (2022) 21:103074. doi: 10.1016/j.autrev.2022.103074, PMID: 35247644

[12] Zhou Y Huang P Huang Z Peng Y Zheng Y Yu Y. Urine cytological study in patients with clinicopathologically confirmed neuronal intranuclear inclusion disease. Front Aging Neurosci. (2022) 14:977604. doi: 10.3389/fnagi.2022.977604, PMID: 36172483 PMC9510843

[13] Sone J Mori K Inagaki T Katsumata R Takagi S Yokoi S. Clinicopathological features of adult-onset neuronal intranuclear inclusion disease. Brain. (2016) 139:3170–86. doi: 10.1093/brain/aww249, PMID: 27797808 PMC5382941

[14] Tian Y Zhou L Gao J Jiao B Zhang S Xiao Q. Clinical features of NOTCH2NLC-related neuronal intranuclear inclusion disease. J Neurol Neurosurg Psychiatry. (2022) 93:1289–98. doi: 10.1136/jnnp-2022-329772, PMID: 36150844 PMC9685690

[15] Zhu R Qu J Xu G Wu Y Xin J Wang D. Clinical and multimodal imaging features of adult-onset neuronal intranuclear inclusion disease. Neurol Sci. (2024) 45:5795–805. doi: 10.1007/s10072-024-07699-y, PMID: 39023713 PMC11554744

[16] Binks SNM Klein CJ Waters P Pittock SJ Irani SR. LGI1, CASPR2 and related antibodies: a molecular evolution of the phenotypes. J Neurol Neurosurg Psychiatry. (2018) 89:526–34. doi: 10.1136/jnnp-2017-315720, PMID: 29055902 PMC5909759

